# Efficacy and safety of a novel TKI (anlotinib) for the treatment of advanced digestive system neoplasms: a systematic review and meta-analysis

**DOI:** 10.3389/fimmu.2024.1393404

**Published:** 2024-08-14

**Authors:** Changhui Zhou, Weihua Wang, Ying Mu, Min Meng

**Affiliations:** ^1^ Department of Central Laboratory, Liaocheng People’s Hospital, Liaocheng, Shandong, China; ^2^ Department of Gastroenterology, Liaocheng People’s Hospital, Liaocheng, Shandong, China

**Keywords:** anlotinib, target therapy, conventional treatment, digestive system neoplasms, meta-analysis

## Abstract

**Objective:**

To systematically evaluate the efficacy and safety of anlotinib targeted therapy for the treatment of patients with advanced digestive system neoplasms (DSNs).

**Methods:**

Clinical trials were extracted from PubMed, the Cochrane Library, Web of Science, Embase, China National Knowledge Infrastructure (CNKI) and the Wanfang database up to October 2023. Outcome measures, including therapeutic efficacy, quality of life (QOL) and adverse events, were extracted and evaluated.

**Results:**

Twenty trials, including 1,613 advanced DSNs patients, were included. The results indicated that, compared with conventional treatment alone, the combination of anlotinib targeted therapy with conventional treatment significantly improved the patients’ 6-months overall survival (OS, OR=1.76, CI=1.53 to 2.02, *P*<0.00001), overall response (ORR, OR=1.76, CI=1.53 to 2.02, *P*<0.00001) and disease control rate (DCR, OR=1.51, 95% CI=1.25 to 1.84, *P*<0.0001). Moreover, the group that received the combined therapy had higher rates of hypertension (*P*<0.00001), proteinuria (*P*<0.00001), fatigue (*P*<0.00001), diarrhea (*P*<0.00001), hypertriglyceridemia (*P*=0.02), alanine aminotransfease (ALT)increased (*P*=0.004), aspartate transaminase (AST) increased (*P*=0.006), anorexia (*P*<0.00001), weight loss (*P*=0.002), abdominal pain (*P*=0.0006), hypothyroidism (*P*=0.02), prolonged QT interval (*P*=0.04). Analyses of other adverse events, such as gastrointestinal reaction, leukopenia, and neutropenia, did not reveal significant differences (*P*>0.05).

**Conclusion:**

The combination of anlotinib targeted therapy and conventional treatment is more effective for DSNs treatment than conventional treatment alone. However, this combined treatment could lead to greater rates of hypertension, albuminuria and hand-foot syndrome. Therefore, the benefits and risks should be considered before treatment.

## 1 Introduction

Digestive system neoplasms (DSNs) are an important part of the incidence and mortality rate of cancer in the world, and cause 3,524,932 deaths in 2020, which accounts for 18% of all cancer deaths worldwide (1–3). This category comprises colorectal cancer, gastric cancer, liver cancer, esophageal cancer, and pancreatic cancer, which are the third, sixth, seventh, tenth, and fourteenth most common cancers, respectively (4). Gastrointestinal malignant tumor is a common tumor of the digestive system in the clinic, which threatens the human’s life and health seriously (5). The three main modalities (chemotherapy, targeted therapy and immunotherapy) had been widely used in treating patients with DSNs (6). Despite the extraordinary improvements carried out in diagnostic and therapeutic management of DSNs in the past few decades, the 5-year survival rate of patients is still very low (1, 7). Since DSNs are mostly detected only at advanced stages, early extensive invasion and distant metastasis, as well as a profound resistance towards multi-drugs contribute to poor prognosis for the patients (8–10). Therefore, the effective and new therapeutic strategies targeting DSNs should be developed.

In recent years, molecular-targeted agents have attracted substantial attention to improve the anti-cancer specificity and efficacy and significantly reduce non-selective resistance and toxicity (11). Targeted therapy is a type of cancer treatment that uses drugs or other substances by targeting cancer-specific genes, proteins, or the tissue environment that control cancer cells’ growth, division and spreading (12, 13). Compared to traditional chemotherapy drugs, targeted anti-tumor drugs can specifically act on cancer cells with high efficacy and little damage is done to normal cells (14). As a result of the rapid innovations and advancements in the field of tumor biology, more and more attention has been focused on the new modality of tumor molecular-targeted therapy for advanced cancer (11). Multiple clinical studies have confirmed that molecular targeted therapy combined with conventional treatment methods has better effects on cancer patient (15–18).

Over the past few decades, increasing evidence has indicated the important role of neovascularization in proliferation, migration, and invasion of various solid tumors (19). Vascular endothelial growth factor (VEGF), fibroblast growth factor, platelet-derived growth factor and their corresponding receptors play an important role in the process of vascular growth. Therefore, vascular-targeted therapy against these growth factors and their receptors is one of the important strategies for patients with advanced DSNs. Anlotinib is a novel and oral small-molecule multitarget tyrosine kinase inhibitor (TKI), which is able to inhibit both tumor angiogenesis and proliferation by targeting vascular endothelial growth factor receptor (VEGFR) 1/2/3, stem cell-factor receptor, platelet-derived growth factor receptors (PDGFR)-α, and fibroblast growth factor receptor (FGFR) 1/2/3 (20, 21). Anlotinib has now been approved for the treatment of lung cancer, soft tissue sarcoma, and other solid tumors (21, 22). It was independently developed by Chia Tai Tianqing Pharmaceutical Group, and has been approved by the China National Medical Products Administration for patients in China since May 2018. In several clinical trials, anlotinib therapy combined with conventional chemotherapy exhibited more prominent therapeutic effects for patients with advanced DSNs than conventional treatment alone (23–25). However, systematic review of clinical trials assessing the therapeutic efficacy of anlotinib in combination with chemotherapy in advanced DSNs patients remains scarce.

In this study, we conducted a systematic review and meta-analysis to investigate the efficacy and safety of combined use of anlotinib with conventional chemotherapy in patients with advanced DSNs to provide a scientific reference for the design of future clinical trials.

## 2 Methods

This meta-analysis was performed in accordance with the Preferred Reporting Items for Systematic Reviews and Meta-Analyses guidelines (26). No further ethical approval is required since the program does not require the recruitment of patients and the collection of personal information.

### 2.1 Search strategy

Related Literatures were searched across nine electronic databases, including Cochrane Library, Web of Science, Embase, Medline, PubMed, Chinese Scientific Journal Database (VIP), Wanfang database, Chinese Biological Medicine Database (CBM) and China National Knowledge Infrastructure (CNKI). Publications in English and Chinese dated from the inception of the database to October 2023 were shortlisted using the following search terms: “anlotinib” combined with “gastric cancer” or “colorectal cancer” or “gastrointestinal cancer” or “liver cancer” or “esophageal cancer” or “pancreatic cancer” or “digestive system neoplasms” without restriction on the language.

### 2.2 Eligibility criteria

#### 2.2.1 Inclusion criteria

Inclusion criteria for this review were (1): Randomized controlled trials (RCTs) concerning DSNs patients were included; (2) Patients are diagnosed as DSNs by pathology. The nationality, race, gender, and age of the patients included in the study are not limited; (3) Articles involving more than 40 DSNs patients; (4) Literatures comparing the clinical outcomes of regular treatments plus anlotinib targeted therapy (experimental group) with regular treatments alone (control group); (5) Overall response rate (ORR), disease control rate (DCR) and treatment–related adverse effects must be included in each study.

#### 2.2.1 Exclusion criteria

Exclusion criteria were: (1) Studies not focus on anlotinib were excluded; (2) Inappropriate criteria in experimental or control group were excluded; (3) Articles without sufficient available data were excluded; (4) Non-RCTs, literature reviews, meta-analysis, meeting abstracts, case reports, repeated studies and experimental model researches were excluded.

### 2.3 Quality assessment

To ensure the quality of the meta-analysis, the quality of the included RCTs was evaluated according to the Cochrane Handbook tool (27).

### 2.4 Types of outcome measures

The primary outcomes in present analysis included short-term and long-term clinical efficacy, and adverse effects (AEs) according to the World Health Organization criteria and Response Evaluation Criteria in Solid Tumors 1.1 (RECIST Criteria 1.1) (28). The primary outcomes were: (1) Short-term clinical efficacy: the short-term tumor response included overall response rate (ORR, the sum of complete response and partial response) and disease control rate (DCR, the sum of complete response, partial response and stable disease); (2) Long-term clinical efficacy: 1-5 year overall survival (OS) defined as the time from the date of randomization to death from any cause; (3) Treatment–related adverse effects; (4) Quality of life (QOL): QOL was evaluated using Karnofsky score.

### 2.5 Data extraction and management

The following data were extracted from eligible studies: (1) Study characteristics such as name of the first author, patient ages, year of publication, number of cases, and study parameter types; (2) Details of the interventions such as intervention technique as well as dosage, administration route, and duration of anlotinib treatment; (3) Outcomes measures and other parameters that included the OS, ORR, DCR, Karnofsky performance score (KPS), and AEs. We attempted to contact the authors to request missing or incomplete data. If the relevant data could not be acquired, the studies were excluded from the analysis.

### 2.6 Statistical analysis

Stata 16.0 (Stata Corp., College Station, TX, USA) and Review Manager 5.3 (Nordic Cochran Centre, Copenhagen, Denmark) statistical software were used for statistical analyses. Dichotomous data were represented by the risk ratio (RR) with the respective 95% confidence interval (CI), whereas continuous variables were expressed as mean difference (MD) with 95% CI. *P*<0.05 indicates difference with statistical significance. Heterogeneity among studies was estimated using the Cochran’s Q statistic and *I^2^* tests, and *I^2^* > 50% or *P*<0.1 indicated a high statistical heterogeneity (29). A fixed-effects model was used to pool the estimates when heterogeneity was absent (*I^2^* < 50%). Otherwise, a random effects model was selected.

Any publication bias was investigated using funnel plots and the Begg and Egger tests for parameters that were reported in more than 10 studies (30–32). A trim-and-fill method was used to coordinate the estimates from unpublished studies if publication bias existed, and the adjusted results were compared with the original pooled RR (33). Subgroup analysis was conducted to investigate the influence of cancer types, and therapeutic regimens.

## 3 Results

### 3.1 Search results

**Figure 1:**
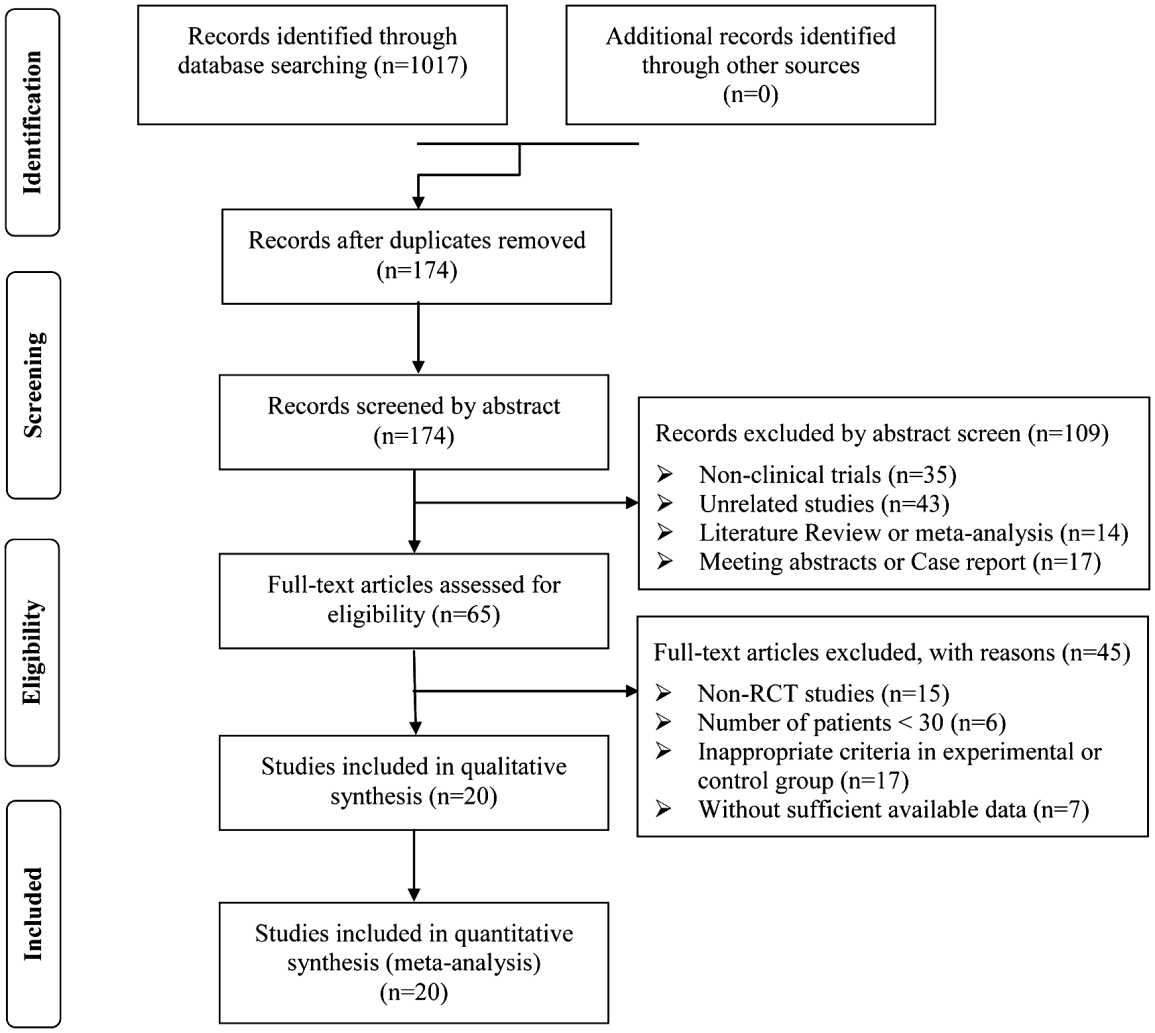
Flow diagram of the selection process.

A total of 1,017 articles were identified with initial retrieve. 843 papers were excluded due to duplication. After title and abstract review, 109 articles were further excluded because they were not clinical trials (n=35) or were unrelated studies (n=43) or were literature review and meta-analysis (n=14) or were meeting abstract and case report (n=17), leaving 65 studies as potentially relevant. After detailed assessment of full texts, articles were not RCTs (n=15), studies with a sample size of less than 30 (n=6); publications with inappropriate criteria of experimental or control group (n=17), and trials with insufficient data (n=7) were excluded. Finally, 20 trials (23, 25, 34–51) involving 1,613 DSNs patients were included in this analysis (**Figure 1**).

### 3.2 Patient characteristics

In total, 934 DSNs patients were treated by regular treatments in combination with anlotinib targeted therapy, while 679 patients were treated by regular treatments alone. Detailed information of the involved studies and DSNs patients is shown in **Tables 1**, **2**. All included trials except one (49) clearly introduce the dosage and duration of anlotinib treatment.

**Table 1:** Clinical information from the eligible trials in the meta-analysis.

Included studies	Tumor type	Tumor stage	Patients Con/Exp	Age (year)		Parameter types
				Control group	Experimental group	
Chi Y 2021 (23)	CC	TNM (IV)	137/282	55.2±10.8 (mean)	56.2 ±10.5 (mean)	OS, ORR, DCR, AE
Dai KJ 2022 (34)	CC	NK	44/44	51.31 ± 8.64 (mean)	50.79 ± 9.19 (mean)	ORR, DCR, AE
Huang J 2020	EC	TNM (IV)	55/109	45–76	43–74	OS, AE
Lan L 2020 (35)	GC	TNM (II-IV)	20/60	43-72	41-73	OS, ORR, DCR, AE
Liu L 2023 (36)	HC	BCLC (B/C)	40/40	54.12±8.95 (mean)	55.28±8.42 (mean)	ORR, DCR, KPS, AE
Liu YJ 2021 (37)	EC	TNM (II/III)	25/23	48.58 ± 2.35	48.26 ± 2.62	OS, ORR, DCR, AE
Lu JY 2021 (38)	HC	Advanced Stage	30/30	64.37±3.19 (mean)	64.63±3.82 (mean)	ORR, DCR, AE
Pang H 2022 (39)	EC	Advanced Stage	28/29	NK	NK	ORR, DCR, KPS, AE
Pei SF 2023 (40)	EC	TNM (III/IV)	53/53	53.6±6.8 (mean)	54.1±7.2 (mean)	OS, ORR, DCR, AE
Wang C 2020 (41)	EC	TNM (IV)	30/30	≥60 25	≥60 22	ORR, DCR, AE
Wang ZY 2019 (42)	EC	TNM (II/III)	18/18	49.1 ±7.3	48.3±8.4	OS, ORR, DCR, AE
Xiong HP 2022 (43)	EC	Advanced Stage	20/21	57.69±6.52	58.10±6.78	ORR, DCR, AE
Xu YW 2021 (44)	EC	TNM (II/III)	34/34	52.36±5.74	52.69±5.58	AE
Xue WL 2019 (45)	GC	TNM (III/IV)	18/18	55.2±2.5	54.7±2.3	ORR, DCR
Xue WL 2020a (46)	EC	TNM (II-IV)	17/18	52.6±2.9	51.7±3.1	ORR, DCR, KPS
Xue WL 2020b (47)	CC	NK	17/17	51.3±3.2 (mean)	50.9±3.1 (mean)	ORR, DCR, AE
Yang WW 2022 (48)	CC	Advanced Stage	19/34	<65 (89.47)	<65 (88.24)	OS, ORR, DCR, AE
Zhang XW 2020 (49)	EC	Advanced Stage	28/28	59.36±7.10	59.64±7.01	OS, ORR, DCR
Zhao HJ 2021 (50)	EC	TNM (IV)	26/26	53.72±9.81	55.68±11.76	ORR, DCR, KPS, AE
Zhou CS 2022 (51)	GC	TNM (III/IV)	20/20	72.1±2.8	74.3±3.3	ORR, DCR, KPS, AE

Control group, Conventional treatment group; Experimental group, Anlotinib combined conventional treatment group.

CC, colorectal cancer; EC, esophageal cancer; HC, Hepatocellular cancer; GC, gastric cancer; TNM, tumor node metastasis classification; BCLC, Barcelona clinic liver cancer staging classification;  NK, unknown; KPS, karnofsky performance score; OS, Overall survival; ORR, overall response rate; DCR, disease control rate; AE, adverse events.

**Table 2:** Information of anlotinib combined with conventional treatment.

Included studies	Therapeutic regimen		Enrollment Period
	Experimental group	Control group	
Chi Y 2021 (23)	Anlotinib (12mg per d, per os; d 1-14; 21 days per cycle [a, b, c])	Placebo	2014.12-2016.8
Dai KJ 2022 (34)	Anlotinib (12mg per d, per os; d 1-14; 21 days per cycle; 2 cycles)+capecitabine.	Capecitabine (1250 mg/m^2^)	2018.9-2020.3
Huang J 2020	Anlotinib (12mg per d, per os; d 1-14; 21 days per cycle [a, d])	Placebo	2016.1-2018.5
Lan L 2020 (35)	Anlotinib (12mg per d, per os; d 1-14; 21 days per cycle [b])	Placebo	2015.2-2016.5
Liu L 2023 (36)	Anlotinib (12mg per d, per os; d 1-14; 21 days per cycle)+ TACE.	TACE (Epirubicin, 10mg; Oxaliplatin, 50mg)	2020.1-2022.1
Liu YJ 2021 (37)	Anlotinib (12mg per d, per os; d 1-14; 21 days per cycle; 2 cycles) + Chemotherapy + Radiotherapy	Chemotherapy (Paclitaxel, 50 mg//m^2^; Carboplatin, AUC = 2) + Radiotherapy (1.8-2.0 Gy/d 5 days per week; 54-60 Gy in total)	2018.9-2020.9
Lu JY 2021 (38)	Anlotinib (12mg per d, per os; d 1-14; 21 days per cycle [a])+TACE.	TACE	2014.1-2016.1
Pang H 2022 (39)	Anlotinib (8mg per d, per os; d 1-14; 21 days per cycle [a])+ Radiotherapy.	Radiotherapy (2.0 Gy/d 5 days per week; 60 Gy in total)	2019.7-2021.7
Pei SF 2023 (40)	Anlotinib (12 mg per d, per os; d 1-14; 21 days per cycle) + Chemotherapy + Radiotherapy	Chemotherapy (Cisplatin, 80mg/m^2^; 5- Fluorouracil, 1000mg/m^2^) + Radiotherapy (1.8-2.0 Gy/d 5 days per week; 54-60 Gy in total)	2017.1-2019.1
Wang C 2020 (41)	Anlotinib (12 mg per d, per os; d 1-14; 21 days per cycle; 2 cycles [a])+S-1	S-1, 80 mg /m^2^ ·d	2018.6-2019. 9
Wang ZY 2019 (42)	Anlotinib (12 mg per d, per os; d 1-14; 21 days per cycle)+ Radiotherapy.	Radiotherapy (1.8-2.0 Gy/d 5 days per week; 63.4-68 Gy in total)	2017.6-2017.9
Xiong HP 2022 (43)	Anlotinib (12 mg per d, per os; d 1-14; 21 days per cycle [a])+ Camrelizumab	Camrelizumab, 200 mg, once every 3 weeks.	2019.2-2021. 9
Xu YW 2021 (44)	Anlotinib (12 mg per d, per os; d 1-14; 21 days per cycle) + Chemotherapy + Radiotherapy	Chemotherapy (Cisplatin 75mg/m^2^, Fluorouracil 750-1000mg/m^2^) + Radiotherapy (1.8-2.0 Gy/d 5 days per week; 54-60 Gy in total)	2018.1-2019.4
Xue WL 2019 (45)	Anlotinib (12 mg per d, per os; d 1-14; 21 days per cycle; 3 cycles) + Chemotherapy	Chemotherapy (Fluorouracil, 500 mg/m^2^)	2018.1-2019.5
Xue WL 2020a (46)	Anlotinib (12 mg per d, per os; d 1-14; 21 days per cycle; 2 cycles) + Chemotherapy+ Radiotherapy	Chemotherapy (Capecitabine,1000 mg/m^2^ ·d) + Radiotherapy (2.0 Gy/d 5 days per week; 64 Gy in total)	2018.1-2019. 1
Xue WL 2020b (47)	Anlotinib (12 mg per d, per os; d 1-14; 21 days per cycle; 2 cycles) + Chemotherapy	Chemotherapy (Capecitabine,2500 mg/m^2^ ·d)	2018.1-2019.1
Yang WW 2022 (48)	Anlotinib (12mg per d, per os; d 1-14; 21 days per cycle [a, b, c]) + Chemotherapy	Chemotherapy (Fluorouracil, Oxaliplatin/Irinotecan) + Placebo	2014.9-2016.8
Zhang XW 2020 (49)	Anlotinib (ND) + Radiotherapy	Radiotherapy (2.0 Gy/d 5 days per week; 55-65 Gy in total)	2017.1-2018.1
Zhao HJ 2021 (50)	Anlotinib (12 mg per d, per os; d 1-14; 21 days per cycle; 2 cycles) + Chemotherapy	Chemotherapy (Irinotecan 125mg /m^2^)	2018.10-2018.10
Zhou CS 2022 (51)	Anlotinib (12 mg per d, per os; d 1-14; 21 days per cycle; 3 cycles) + S-1	S-1 (40-60 mg/time, 2 times/day)	2018.10-2020.10

Control group, Conventional treatment group; Experimental group, Anlotinib combined conventional treatment group; a: The treatment continued until PD or intolerable toxicity; b: If the patient could not tolerate 12mg/day, then the dose could be reduced to 10 mg/day or 8 mg/day; c: If the dose of 8 mg/day was not tolerated, then treatment was terminated in accordance with the RECIST; d: Treatment interruptions and dose modifications due to treatment-related toxicities were allowed.

TACE, Transcatheter hepatic arterial chemoembolization; PD, Progressive disease; NK, unknown; S-1, Gimeracil and Oteracil Porassium Capsules.

### 3.3 Quality assessment

**Figure 2:**
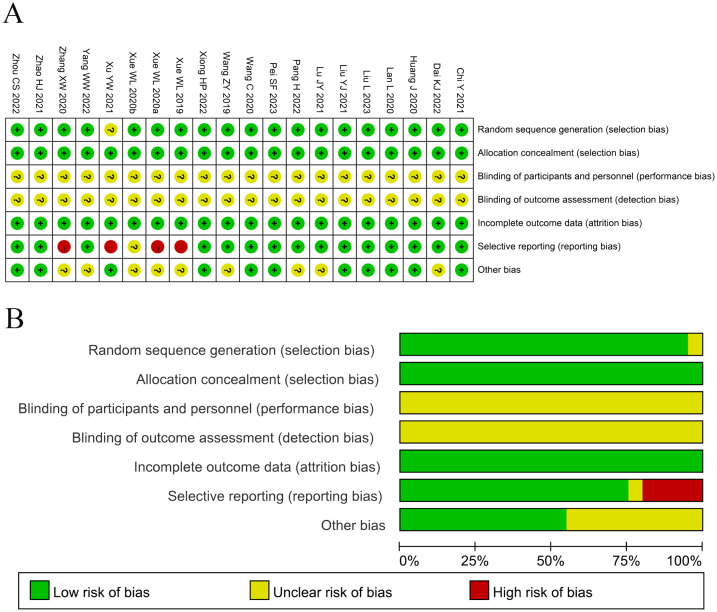
**(A)** Risk of bias summary: Review of the authors’ judgments about each risk of bias item for the included studies. **(B)** Risk of bias graph: Review of the authors’ judgments about each risk of bias item presented as percentages across all included studies. Each color represents a different level of bias: red for high-risk, green for low-risk, and yellow for unclear risk of bias.

The assessment of bias risk is shown in **Figure 2**. Among the studies involved in the present analysis, nineteen were determined to have a low risk of bias and the remaining one did not offer a clear description of the randomization process. The selection and attrition risks of involved trials were low. None of the trials included in the present analysis provided a clear description of the performance and detection risks. Among the trials, one were considered to present unclear risk, owing to selective reporting, whereas four studies were considered as high risk, on account of the lack of data pertaining to the primary outcome measures.

### 3.4 OS assessments

**Figure 3:**
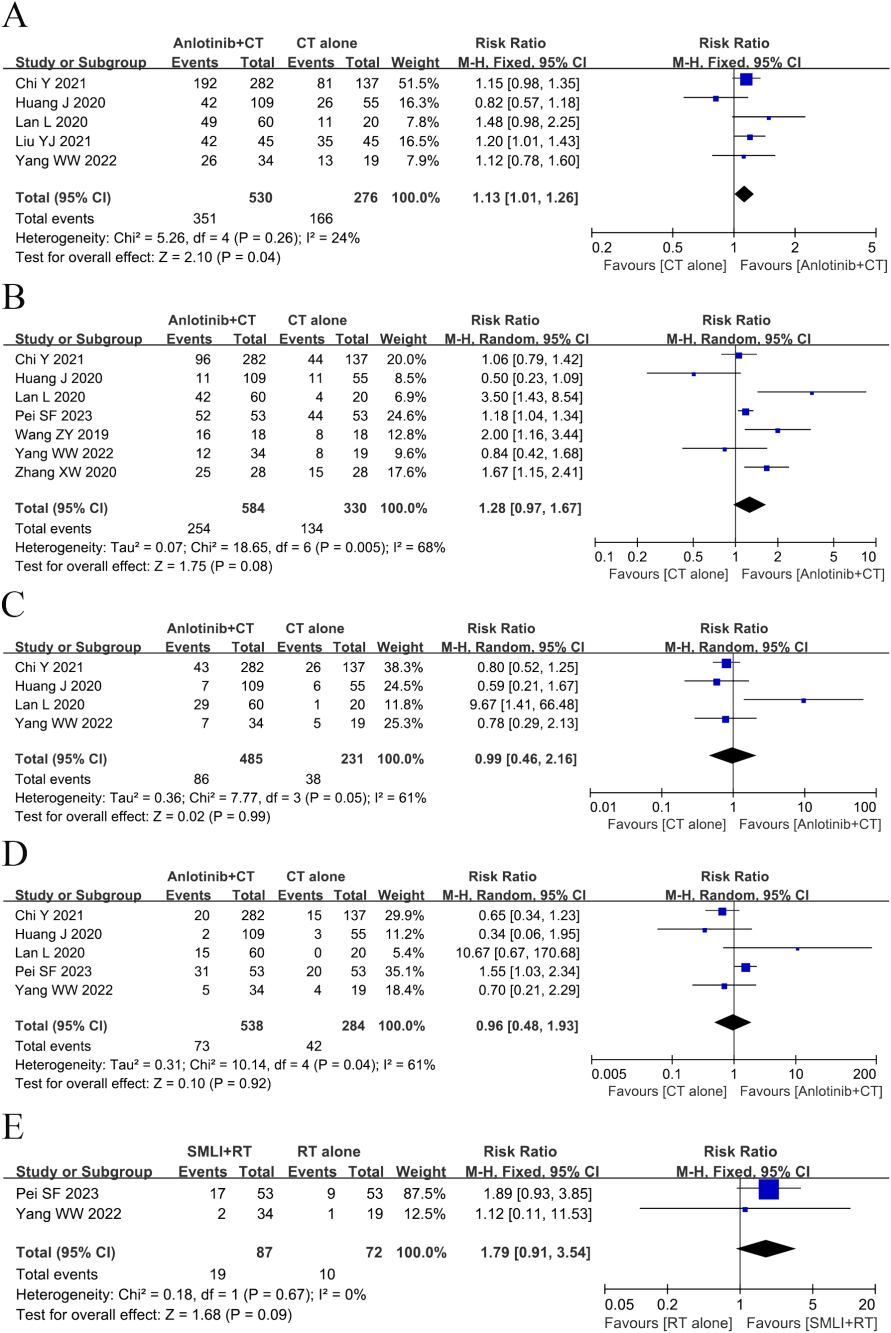
Forest plot of the comparison of the overall survival (OS) between the experimental and control group. **(A)** 6-months OS, **(B)** 12-months OS, **(C)** 18-months OS, **(D)** 24-months OS, and **(E)** 36-months OS. Control group, conventional treatment group; experimental group, anlotinib combined conventional treatment group; CI, confidence interval. The fixed-effects meta-analysis model (Mantel–Haenszel method) was used.

Eight clinical trials (23, 25, 35, 37, 40, 41, 48, 49) involving 1,004 cases compared the OS between the two groups (**Figure 3**). The analysis result of OS was shown in **Figure 3A**. Compared with regular treatments, the combination of regular treatments and anlotinib can increase 6-, 12-, 18-, 24-, and 36-months OS, but only 6-months OS reaches a significant level (6-months OS: RR=1.13, 95% CI=1.01-1.26, *P*=0.04; 12-months OS: RR=1.28, 95% CI=0.97-1.67, *P*=0.08; 18-months OS: RR=0.99, 95% CI=0.46-2.16, P=0.99; 24-months OS: RR=0.96, 95% CI=0.48-1.93, *P*=0.92; 36-months OS: RR=1.79, 95% CI=0.91-3.54, *P*=0.09). 12-, 18-, and 24-months OS (12-months OS: *P*=0.005, *I^2^* = 68%; 18-months OS: *P*= 0.05, *I^2^* = 61%; 24-months OS: *P*=0.04, *I^2^* = 61%) displayed statistical heterogeneity, as per the heterogeneity test. Hence, a random-effects model was used in the meta-analysis. Otherwise, the fixed-effect model was used in case of 6- and 36-months OS.

### 3.5 ORR and DCR assessments

**Figure 4:**
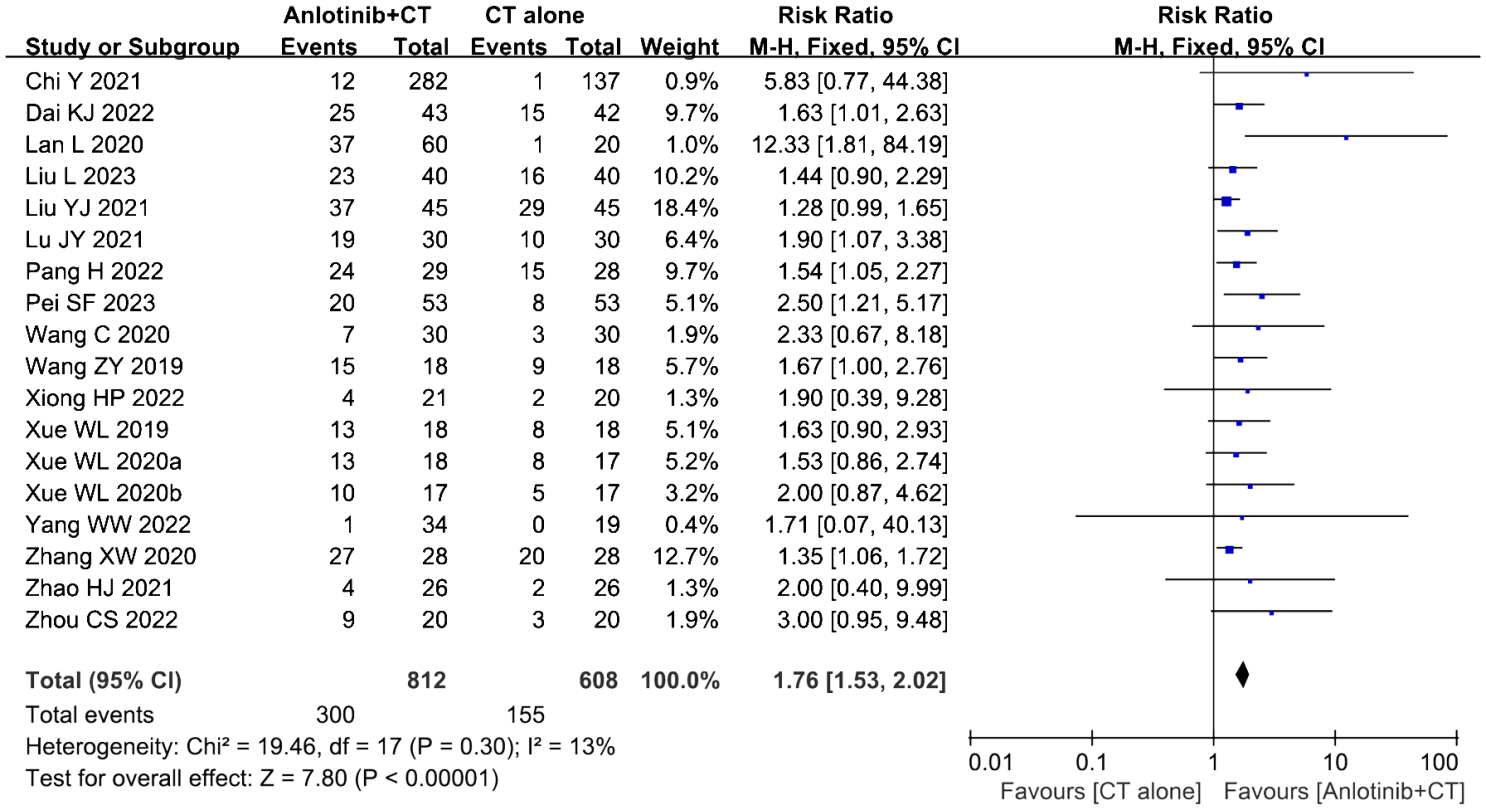
Forest plot of the comparison of the overall response rate (ORR) between the experimental and control group. Control group, conventional treatment group; experimental group, anlotinib combined conventional treatment group; CI, confidence interval. The fixed-effects meta-analysis model (Mantel–Haenszel method) was used.

**Figure 5:**
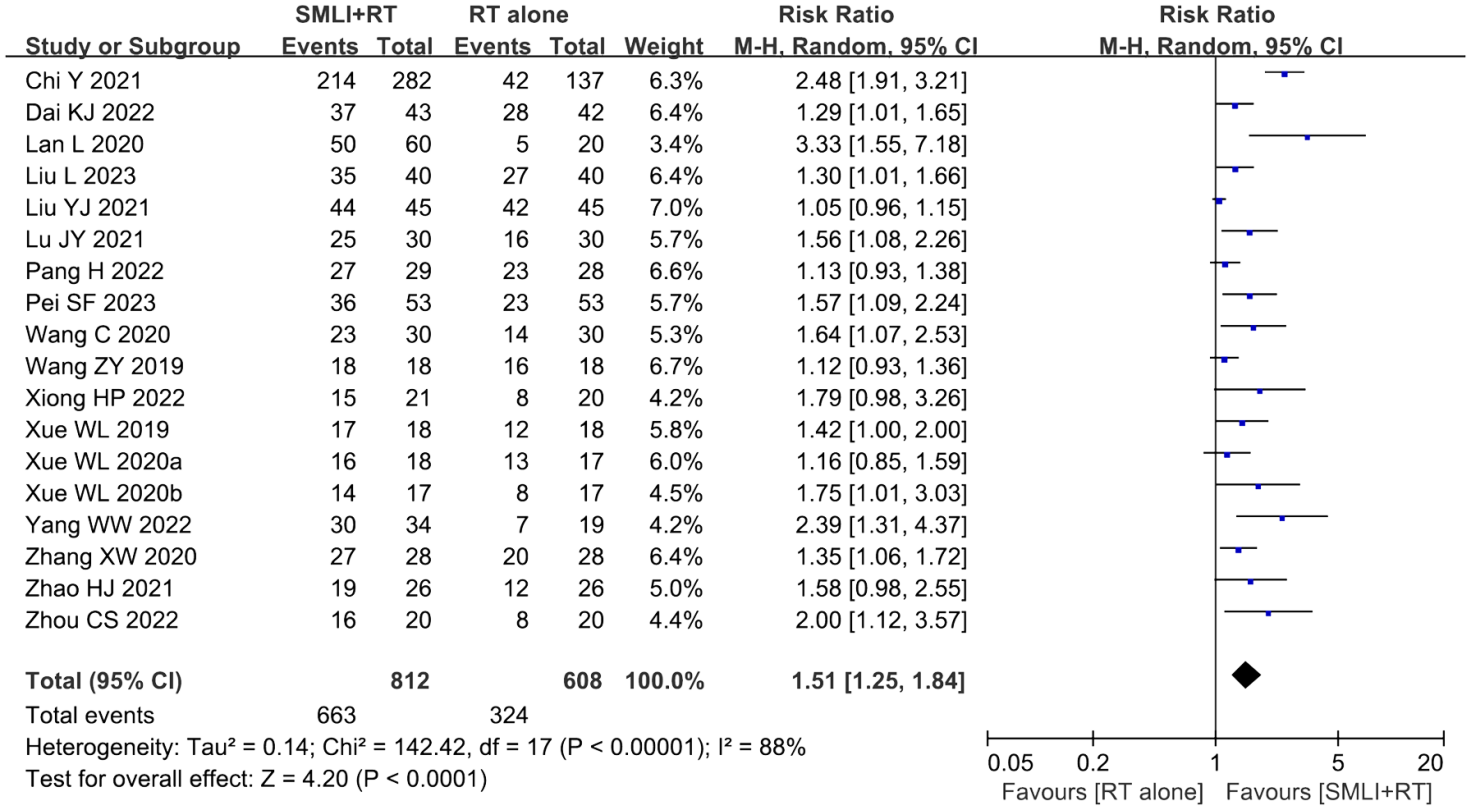
Forest plot of the comparison of the disease control rate (DCR) between the experimental and control group. Control group, conventional treatment group; experimental group, anlotinib combined conventional treatment group; CI, confidence interval. The fixed-effects meta-analysis model (Mantel–Haenszel method) was used.

Eighteen clinical trials (23, 34–43, 45–51) involving 1,420 cases compared the ORR and DCR between the two groups (**Figures 4**, **5**). Our pooled results showed that patients underwent combined therapy had significantly improved ORR and DCR (ORR: RR=1.76, 95% CI=1.53-2.02, *P*<0.00001; DCR: RR=1.51, 95% CI =1.25-1.84, *P*<0.0001) compared with regular treatments alone. DCR (*P*= 0.30, *I^2^* = 13%) displayed slightly significant heterogeneity, as per the heterogeneity test. Hence, a fixed-effect model was used in the meta-analysis. Otherwise, the random-effects model was used in case of DCR.

### 3.6 KPS score

**Figure 6:**
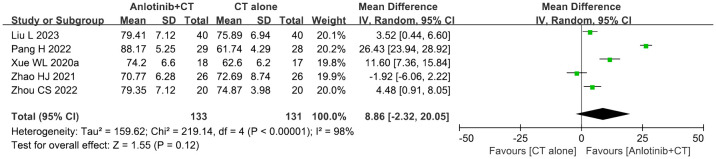
Forest plot of the comparison of the Karnofsky performance score (KPS) between the experimental and control groups. Control group, conventional treatment group; experimental group, anlotinib combined conventional treatment group; CI, confidence interval. The fixed-effects meta-analysis model (Mantel–Haenszel method) was used.

Five trials (36, 39, 46, 50, 51) involving 264 DSNs patients evaluated the QOL according to the KPS Score. As shown in **Figure 6**, the KPS score of DSNs patients in the combined group were higher than that of the control group, but the difference was not statistically significant (MD = 8.86, 95% CI = -2.32-20.05, *P*=0.12). *P*<0.00001 and *I^2^* = 98% indicated that there was significant heterogeneity among the studies; thus a random effect model was employed.

### 3.7 Adverse events assessment

Seventeen trials (23, 25, 34–44, 47, 48, 50, 51) involving 1,486 DSNs patients evaluated the safety of anlotinib mediated therapy. As shown in **Table 3**, the patients who underwent combination therapy exhibited higher incidences of hypertension (RR=2.53, 95% CI=1.87 to 3.41, *P*<0.00001), proteinuria (RR=2.15, 95% CI=1.63 to 2.82, *P*<0.00001), fatigue (RR=1.69, 95% CI=1.40 to 2.04, *P*<0.00001), diarrhea (RR=2.68, 95% CI=1.90 to 3.77, *P*<0.00001), hypertriglyceridemia (RR=1.96, 95% CI=1.10 to 3.47, *P*=0.02), ALT increased (RR=1.93, 95% CI=1.23 to 3.03, *P*=0.004), AST increased (RR=1.74, 95% CI=1.17 to 2.57, *P*=0.006), anorexia (RR=2.23, 95% CI=1.62 to 3.08, *P*<0.00001), weight loss (RR=3.32, 95% CI=1.53 to 7.18, *P*=0.002), abdominal pain (RR=2.50, 95% CI=1.48 to 4.24, *P*=0.0006), hypothyroidism (RR=4.60, 95% CI=1.30 to 16.27, *P*=0.02), and prolonged QT interval (RR=1.67, 95% CI=1.03 to 2.71, *P*=0.04) compared to the patients who underwent conventional therapy. The analysis of gastrointestinal reaction (RR=1.18, 95% CI=0.97 to 1.42, *P*=0.09), leukopenia (RR=1.41, 95% CI=0.94 to 2.09, *P*=0.09), neutropenia (RR=1.39, 95% CI=0.52 to 3.71, *P*=0.52), hemoglobinopenia (RR=0.77, 95% CI=0.48 to 1.22, *P*=0.26), thrombocytopenia (RR=1.15, 95% CI=0.48 to 2.74, *P*=0.75), vomiting and Nausea (RR=1.18, 95% CI=0.81 to 1.72, P=0.39), hypercholesterolemia (RR=1.26, 95% CI=0.90 to 1.77, *P*=0.17), hand-foot syndrome (RR=2.98, 95% CI=0.60 to 14.78, *P*=0.18), oropharyngeal pain (RR=1.30, 95% CI=0.83 to 2.03, *P*=0.25), hepatic function damage (RR=1.21, 95% CI=0.86 to 1.69, *P*=0.28), myelosuppression (RR=1.39, 95% CI=0.83 to 2.35, *P*=0.21), and Rash (RR=1.97, 95% CI=0.70 to 5.58, *P*=0.20) did not reveal any significant difference between the two groups. The incidence of neutropenia (*P*=0.09, *I^2^* = 50%), hypertriglyceridemia (*P*=0.10, *I^2^* = 51%), hand-foot syndrome (*P*=0.09, *I^2^* = 54%), myelosuppression (*P*=0.03, *I^2^* = 63%), hypothyroidism (*P*=0.003, *I^2^* = 79%) and rash (*P*=0.05, *I^2^* = 58%) showed mid to high level heterogeneity, as per the heterogeneity test. Consequently, a random-effects model was used to pool the results in the present meta-analysis. Otherwise, the fixed-effect model was used.

**Table 3:** Comparison of adverse events between the experimental and control group.

Adverse events	Experimental group	Control group	Analysis method	Heterogeneity		Risk Ratio (RR)	95% CI	*P*-value
	No. patients (n)	No. patients (n)		I^2^ (%)	*P*-value			
Hypertension	413	302	Fixed	45	0.06	2.53	1.87-3.41	<0.00001
Proteinuria	635	419	Fixed	0	0.52	2.15	1.63-2.82	<0.00001
Gastrointestinal reaction	236	233	Fixed	3	0.40	1.18	0.97-1.42	0.09
Leukopenia	266	197	Fixed	35	0.17	1.41	0.94-2.09	0.09
Neutropenia	246	177	Random	50	0.09	1.39	0.52-3.71	0.52
Hemoglobinopenia	385	240	Fixed	0	0.70	0.77	0.48-1.22	0.26
Thrombocytopenia	94	79	Fixed	0	0.98	1.15	0.48-2.74	0.75
Fatigue	545	329	Fixed	0	0.85	1.69	1.40-2.04	<0.00001
Diarrhea	578	324	Fixed	6	0.38	2.68	1.90-3.77	<0.00001
Vomiting and Nausea	562	308	Fixed	0	0.86	1.18	0.81-1.72	0.39
Hypertriglyceridemia	451	237	Random	51	0.10	1.96	1.10-3.47	0.02
Hypercholesterolemia	451	237	Fixed	0	0.69	1.26	0.90-1.77	0.17
ALT increased	425	211	Fixed	0	0.49	1.93	1.23-3.03	0.004
AST increased	425	211	Fixed	13	0.32	1.74	1.17-2.57	0.006
Anorexia	465	251	Fixed	27	0.25	2.23	1.62-3.08	<0.00001
Weight loss	391	192	Fixed	0	0.89	3.32	1.53-7.18	0.002
Hand-foot syndrome	129	128	Random	54	0.09	2.98	0.60-14.78	0.18
Oropharyngeal pain	136	135	Fixed	0	0.55	1.30	0.83-2.03	0.25
Abdominal pain	425	211	Fixed	7	0.34	2.50	1.48-4.24	0.0006
Hepatic function damage	212	171	Fixed	0	0.48	1.21	0.86-1.69	0.28
Myelosuppression	172	171	Random	63	0.03	1.39	0.83-2.35	0.21
Hypothyroidism	451	237	Random	79	0.003	4.60	1.30-16.27	0.02
Rash	521	306	Random	58	0.05	1.97	0.70-5.58	0.20
Prolonged QT interval	425	211	Fixed	0	0.90	1.67	1.03-2.71	0.04

Control group, Conventional treatment group; Experimental group, Anlotinib combined conventional treatment group.

### 3.8 Publication bias

Publication bias was assessed by Begg’s and Egger’s regression tests, and was detected in indicators such as ORR, DCR and partial side effect indicators (number of included studies > 7). A trim-and-fill analysis was performed, in order to determine whether the publication bias affected the pooled risk. The adjusted RR indicated same trend with the result of the primary analysis, reflecting the reliability of our primary conclusions (**Table 4**).

**Table 4:** Summary of publication bias.

Publication Bias	ORR	DCR	12-OS	Adverse events				
				Hypertension	Diarrhea	Fatigue	Proteinuria	Vomiting and Nausea
**Begg**	0.012	< 0.001	1.000	0.592	0.266	0.072	0.602	0.764
**Egger**	< 0.001	< 0.001	0.698	0.298	0.289	0.359	0.458	0.721
Trim and fill analysis								
before	*P* < 0.001	< 0.001						
after	*P* < 0.001	< 0.001						

OS, Overall survival; ORR, overall response rate; DCR, disease control rate.

### 3.9 Sensitivity analysis

**Figure 7:**
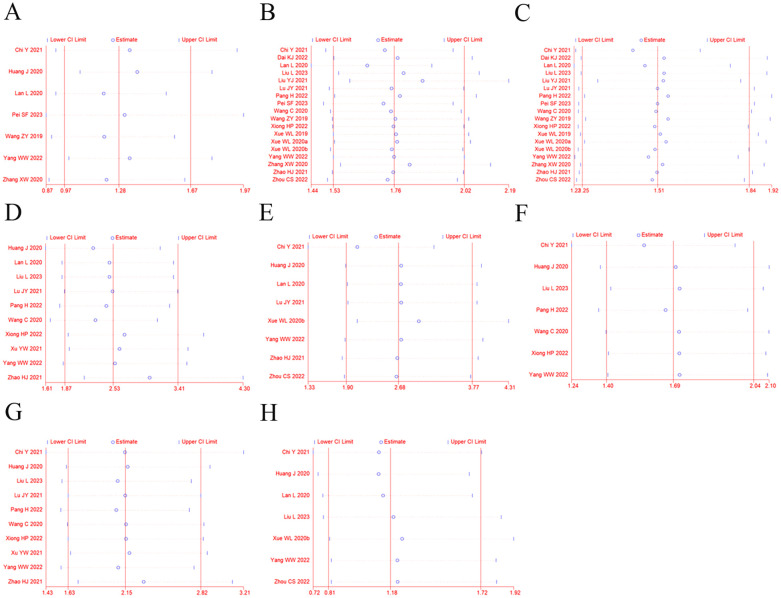
Sensitivity analysis for ORR **(A)**, DCR **(B)**, 12-OS **(C)**, hypertension **(D)**, diarrhea **(E)**, fatigue **(F)**, proteinuria **(G)**, and vomiting and nausea **(H)**.

Sensitivity analysis was performed to explore an individual study’s influence on the pooled results by deleting one single study each time from pooled analysis. As shown in **Figure 7**, the results revealed that none of the individual studies significantly affected the primary outcome measures, which implied statistically robust results.

We also conducted subgroup analysis to explore the source of heterogeneity in ORR and DCR with respect to cancer types, and therapeutic regimens. As shown in **Table 5**, our analysis indicates that the selection of tumor types and the formulation of treatment plans may have a certain impact on the efficacy of anlotinib targeted therapy.

**Table 5:** Subgroup analyses of ORR and DCR between the experimental and control group.

Parameter	Factors at study level	Exp group	Con group	Analysis method	Heterogeneity		Odds Ratio (OR)	95% CI	*P*-value
		No. patients (n)	No. patients (n)		I^2^ (%)	*P*-value			
ORR	Type of cancer								
	Gastric cancer	98	58	Random ndam	72	0.03	3.15	0.91 to 10.86	0.07
	Colorectal cancer	376	215	Fixed	0	0.63	1.97	1.29 to 3.00	0.002
	Hepatocellular Carcinoma	70	70	Fixed	0	0.46	1.62	1.13 to 2.32	0.009
	Esophageal cancer	268	265	Fixed	0	0.66	1.55	1.32 to 1.83	<0.00001
	Therapeutic regimen								
	Anlotinib+Placebo	342	157	Fixed	0	0.59	9.26	2.29 to 37.35	0.002
	Anlotinib+Radiotherapy	75	74	Fixed	0	0.67	1.48	1.21 to 1.82	0.0001
	Anlotinib+Chemotherapy	258	242	Fixed	0	0.98	1.76	1.39 to 2.24	<0.00001
	Anlotinib+Chemoradiotherapy	116	115	Fixed	47	0.15	1.54	1.20 to 1.97	0.0006
DCR	Type of cancer								
	Gastric cancer	98	58	Random	69	0.04	1.98	1.12 to 3.50	0.02
	Colorectal cancer	376	215	Random	82	0.0008	1.88	1.22 to 2.91	0.004
	Hepatocellular Carcinoma	70	70	Fixed	0	0.40	1.40 40	1.13 to 1.72	0.002
	Esophageal cancer	268	265	Fixed	74	0.0001	1.28	1.08 to 1.51	0.004
	Therapeutic regimen								
	Anlotinib+Placebo	342	157	Fixed	0	0.47	2.58	2.01 to 3.30	<0.00001
	Anlotinib+Radiotherapy	75	74	Fixed	0	0.42	1.20	1.06 to 1.36	0.004
	Anlotinib+Chemotherapy	258	242	Fixed	0	0.50	1.54	1.36 to 1.75	<0.00001
	Anlotinib+Chemoradiotherapy	116	115	Random	85	0.002	1.22	0.84 to 1.76	0.29

Control group, Conventional treatment group; Experimental group, Anlotinib combined conventional treatment group.

ORR, overall response rate; DCR, disease control rate.

## 4 Discussion

With the studying development of tumor molecular biology and epigenetic in recent years, increasing numbers of first-line treatment agents, including gefitinib, erlotinib and anlotinib, been suggested for improving therapeutic effects for patients with malignancies (52–54). VEGF is a key mediator of tumor angiogenesis, in which it is up-regulated by oncogene expression, a variety of growth factors and also hypoxia (55). It is essential for endothelial cell functions associated with angiogenesis and plays an important role in angiogenesis, tumor progression and vascular permeability (56, 57). VEGF and their receptors are regarded as the most well-known regulators of neovascularization. VEGF binding to VEGFR provides cell proliferation and vascular tissue formation by the subsequent tyrosine kinase pathway (58). VEGF/VEGFR-related signal pathways leads to endothelial cell differentiation, migration, proliferation, and survival involved in angiogenesis (59). The VEGF/VEGFR system is of great importance in regulating and controlling tumor angiogenesis, and anti-VEGF/VEGFR therapy for cancer are now widely used in the clinical field (60). Researchers have confirmed that the expressions of VEGF and VEGFR signaling pathway exhibited significant correlations with poor prognosis for cancer patients (61–63). Therefore, VEGF/VEGFR axis displays an attractive and potential target for anti-angiogenesis and anti-cancer drug design.

Drugs known as vascular endothelial growth factor receptor tyrosine kinase inhibitor (VEGFR-TKI) can inhibit VEGFR, which have recently been approved and used in treating various cancers, such as renal cell carcinoma (RCC) and liver cancer (64, 65). VEGFR-TKI inhibit angiogenesis induced by tumor cells, leading to the inhibition of cell proliferation and shrinkage of tumors. Thus, VEGFR-TKI are an important option for the treatment of cancer. The VEGFR family includes VEGFR-1 (Flt-1), VEGFR-2 (KDR/Flk-1), VEGFR-3 (Flt-4), and VEGFR co-receptors neuropilin 1 and 2 (66). Among these receptors, VEGFR-2, as an important tyrosine transmembrane protein, is aberrantly expressed in many malignant tumors, and it play an important role in the occurrence, development, and growth of tumors and drug resistance (67). Anlotinib is a novel inhibitor of VEGFR-2 tyrosine kinase with inhibitory effects on angiogenesis and tumor growth, which targets the intracellular ATP binding site of the receptor (68). Several studies have demonstrated that anlotinib has shown good efficacy and tolerability in patients with advanced DSNs (69, 70). Although a number of statistical analyses of clinical trials have been published, the therapeutic and toxic effects have not been systematically demonstrated and evaluated due to the impact of sample size and variability among these clinical trials. Additionally, a variety of different protocols and equation models in these clinical trials may have led to different therapeutic effects. In the present study, an extensive and analytical online search was performed followed by rigorous contrasting and combining analyses to provide a systematical and comprehensive conclusion.

In this study, the efficacy and safety of anlotinib as maintenance therapy for advanced DSNs patients was analyzed and reported from 20 randomized controlled trials. Our meta-analysis revealed that the combined treatment of anlotinib with conventional chemotherapy is associated with a more favorable efficacy compared with conventional treatment alone. The patients who were treated with combined treatment exhibited markedly increased 6-months OS, ORR and DCR (P<0.05). In this analysis, the QOL of patients was also evaluated, and it was found that although the use of anlotinib can improve aspects of quality of life in patients to some extent, but this improvement did not reach a significant difference. These results indicated that the exact efficacy of anlotinib targeted therapy for DSNs patients still needs further research. Safety is the top priority for implementation of clinical treatment, and it is also the key factor for the development of anlotinib targeted therapy. Regarding adverse events and severe toxicities, our analytical results revealed that there were no significant differences in most of the adverse event indicators between the two groups. Consistent with previous reports (22, 25, 71–74), the most common AEs associated with anlotinib are hypertension, proteinuria, loss of appetite, fatigue, diarrhea, dyslipidemia, increased liver transaminase, and hypothyroidism. Most of the AEs are grade 1-2, and only a few patients with grade 3-4 adverse reactions need to reduce the dose of anlotinib, indicating that the side effects of anlotinib were tolerable. All included trials did not report treatment-related deaths. This may indicate that the AE associated with anlotinib is tolerable. To summarize, AEs related to the drug still need to be treated with caution, especially some of the intolerable grade 3 or above AEs. However, on the whole, AEs associated with anlotinib were controllable and the advantages of the use of anlotinib for advanced DSNs outweigh the disadvantages.

Some main factors, such as different treatment regimens and tumor types, may influence the therapeutic effects of anlotinib targeted therapy. The results in our subgroup analysis suggest that anlotinib has a weaker therapeutic effect on patients with advanced gastric cancer compared to other DSNs. However, currently published studies that have probed the influences of these factors on the curative effect of anlotinib targeted therapy are still insufficient. Thus, these issues should be further researched and explored. Furthermore, the determination of the optimal therapeutic strategy will be valuable for DSNs treatment.

There are some limitations in our analysis. First, the number of DSNs patients included in this study is not sufficiently large, and the follow-up time was short. Second, the different trials evaluated the therapeutic efficacy using different outcomes, so it was difficult to summarize the results on the same scale, which led to shrunken statistical sample sizes. Third, our data were partly extracted from published papers rather than original patient records, which mean that we were not able to avoid analytical bias based on the information presented in the articles. Due to the above limitations, future studies and generated data will be valuable to verify the safety and efficacy of anlotinib targeted therapy.

In summary, our study confirmed that the combined treatment of anlotinib with conventional chemotherapy may offer an effective treatment for advanced DSNs patients. Anlotinib targeted therapy markedly enhances the short-term treatment efficacy (ORR and DCR) of conventional treatment for advanced DSNs, but its long-term clinical efficacy remains to be studied further. Moreover, this combined treatment could lead to greater rates of adverse events, such as hypertension, proteinuria and fatigue. Therefore, the potential risks and benefits of treatment options should be considered before treatment.

## Data Availability

The raw data supporting the conclusions of this article will be made available by the authors, without undue reservation.
